# A Rare Atypical Case of Asymptomatic and Spontaneous Intraneural Hematoma of Sural Nerve: A Case Report and Literature Review

**DOI:** 10.1055/a-2218-8461

**Published:** 2024-01-24

**Authors:** Shin Hyuk Kang, Il Young Ahn, Han Koo Kim, Woo Ju Kim, Soo Hyun Woo, Seung Hyun Kang, Soon Auck Hong, Tae Hui Bae

**Affiliations:** 1 Department of Plastic and Reconstructive Surgery, Chung-Ang University Hospital, Dongjak-Gu, Seoul, Republic of Korea; 2 Department of Plastic and Reconstructive Surgery, Chung-Ang University Gwangmyeong Hospital, Gwangmyeong-si, Gyeonggi-do, Republic of Korea; 3 Department of Pathology, Chung-Ang University Hospital, Dongjak-Gu, Seoul, Republic of Korea

**Keywords:** hematoma, intraneural, subparaneurial, sural nerve, case report

## Abstract

Intraneural hematoma is a rare disease that results in an impaired nerve function because of bleeding around the peripheral nerve, with only 20 cases reported. Trauma, neoplasm, and bleeding disorders are known factors for intraneural hematoma. However, here we report atypical features of asymptomatic and spontaneous intraneural hematoma which are difficult to diagnose.

A 60-year-old woman visited our clinic with the complaint of a palpable mass on the right calf. She reported no medical history or trauma to the right calf and laboratory findings showed normal coagulopathy. Ultrasonography was performed, which indicated hematoma near saphenous vein and sural nerve or neurogenic tumor. We performed surgical exploration and intraneural hematoma was confirmed on sural nerve. Meticulous paraneuriotomy and evacuation was performed without nerve injury. Histological examination revealed intraneural hematoma with a vascular wall. No neurologic symptoms were observed.

In literature review, we acknowledge that understanding anatomy of nerve, using ultrasonography as a diagnostic tool and surgical decompression is key for intraneural hematoma. Our case report may help establish the implications of diagnosis and treatment. Also, we suggested surgical treatment is necessary even in cases that do not present symptoms because neurological symptoms and associated symptoms may occur later.

## Introduction

Intraneural hematoma is a rare disease that results in an impaired nerve function because of bleeding around or into the peripheral nerve. 1 Trauma, neoplasm, bleeding disorders, and anticoagulants are known factors that contribute to hemorrhagic neuropathy. It can also be caused by interventional procedures such as fine needle aspiration or nerve blocks. 2 3 4 Most of intraneural hematoma is known to appear on median nerve, but there are few cases from lower extremity nerve. Neuropathic symptoms such as neuropathic pain, paresthesia, and weakness in the innervation area of the affected nerve are common symptoms of intraneural hematoma. 3 5 However, it is difficult to make a diagnosis encountering a patient with atypical clinical features, especially on rare diseases. Here, we report a case of spontaneous and asymptomatic subparaneurial hematoma of the sural nerve in a healthy woman.

## Case

**Fig. 1:**
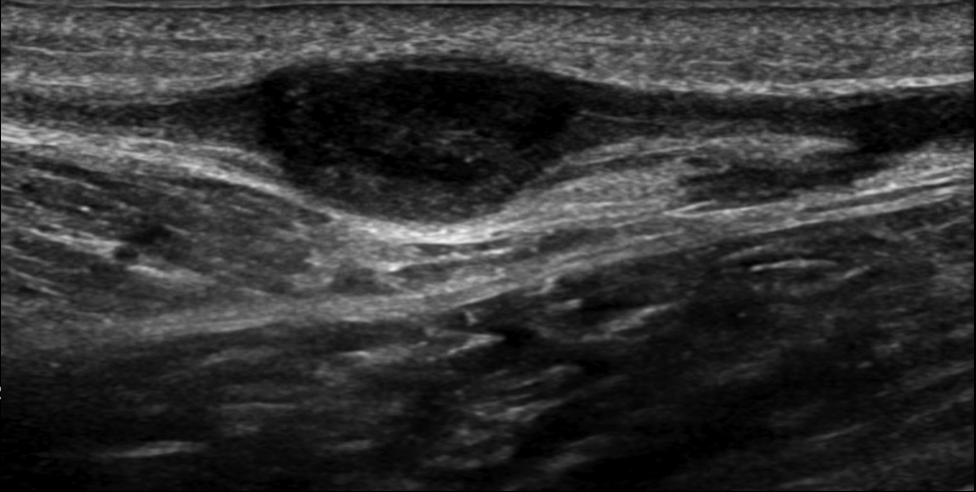
Ultrasonography findings of intraneural hematoma on the right calf.

A 60-year-old woman with a history of osteoarthritis in both knees visited the plastic and reconstructive surgery outpatient clinic with the chief complaint of a palpable mass on the right calf. About 1.5-cm round and hard mass was shown on right calf without skin lesion or distinct symptoms such as pain, paresthesia, or weakness. She reported no history of trauma or medical procedures such as nerve block or fine needle aspiration to the right calf. She had not taken any anticoagulants such as aspirin or warfarin and had no family history on vascular diseases. For preoperative evaluation, ultrasonography and laboratory examinations were performed. Ultrasonography showed a heterogenic hypoechoic mass near the saphenous vein and sural nerve indicating organized thrombus or neurogenic tumor ( Fig. 1 ). No abnormal results were confirmed on laboratory examination including prothrombin time, activated partial thromboplastin time, and international normalized ratio. For possibility of enlargement of mass and neurogenic symptoms, we decided to remove it surgically.

**Fig. 2:**
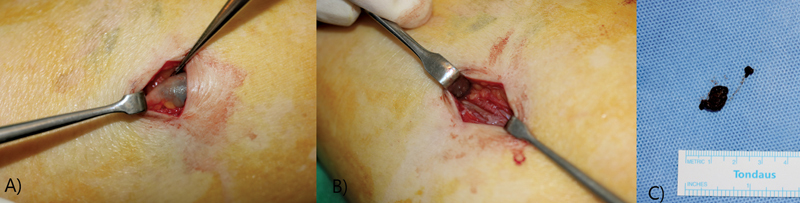
( **A** ) Preoperative findings: subparaneurial hematoma of sural nerve. ( **B** ) Postoperative findings: paraneuriotomy and hematoma evacuation is performed. ( **C** ) Excised hematoma of sural nerve.

With transverse incision, meticulous dissection was performed ensuring no damage to blood vessels and nerves including saphenous vein. After careful dissection, intraneural mass with hematoma formation in sural nerve near the saphenous vein was found. We carefully performed paraneuriotomy with hematoma evacuation using microscissors to prevent damage to the nerve fascicles ( Fig. 2A–C ). No active bleeding was shown. After saline irrigation, layer-by-layer closure was performed.

**Fig. 3:**
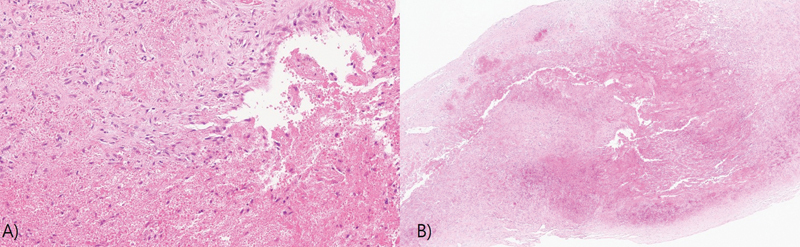
( **A** ) Pathological finding, intraneural hematoma with a vascular wall (×40). ( **B** ) Pathological findings of organized hematoma was observed in high-power view (×200).

Histological examination confirmed an intraneural hematoma with highlighted vascular wall on Masson's trichrome staining ( Fig. 3A, B ). The wound was clean and no neurologic symptoms such as hypoesthesia or weakness of leg were observed.

## Discussion

Intraneural hematoma is a rare disease, with only 20 reported cases in the literature. 6 It is known that upper extremity nerve, especially median nerve is the most common site, but rarely appears in lower extremity nerve. Etiology of intraneural hematoma includes trauma from blunt injury or puncture wound, neoplasm, prolonged anticoagulant therapy, inherited coagulopathy, or interventional procedures such as fine needle aspiration or nerve block. 1 3 4

The case of our patient is rare because she had not consumed any anticoagulants, did not have inherited bleeding disorders, and had no specific traumatic or surgical history. There is a report of spontaneous intraneural hematoma on sural nerve. 7 Since course of sural nerve runs superficial area of posterior leg, there are chances of trauma that patient could not relate with hematoma. Extraneural compression of sural nerve due to direct trauma has been previously reported owing to high chance of trauma on superficial posterior leg. 8 9 Nevertheless, cases of intraneural hematoma are extremely rare with only one case report.

Ultrasonography and magnetic resonance imaging are generally considered the standard imaging modalities for evaluating hemangiomas. 10 Ultrasonography is considered the initial imaging modality of choice. On ultrasonograms, hemangiomas may have a heterogeneous appearance ranging from hypoechoic to hyperechoic relative to the surrounding soft tissue. Intraneural hemangiomas may present as intraneural and circumscribed lesions of varying echogenicity. On magnetic resonance imaging, hemangiomas may have intermediate to decreased signal intensity on T1-weighted images and generally show increased signal intensity on T2-weighted images. Heterogeneous signal intensity can be observed in case of thrombosis or hemorrhage. 6 11 In our case, ultrasonography showed a hypoechoic mass of 1.5 cm on the right calf. Therefore, we considered it an intravenous thrombus or neurogenic hemangioma.

Contrary to other reports about intraneural hematoma, our patient did not present any symptoms such as pain, paresthesia, and numbness. Asymptomatic intraneural hematoma on lower extremity nerve has not been reported to our knowledge. This is probably because the hematoma was identified before it grew larger and compressed the nerve. Surgical treatment is necessary even in cases that do not present symptoms because neurological symptoms and associated symptoms may occur later. If patients refuse surgical treatment, they should be explained that that if repeated pressure is applied to the hematoma, it may grow larger and can press on adjacent nerves, which can cause neurogenic symptoms. Prophylactic surgical treatment might be an option since there are chances of becoming larger mass to avoid associated symptoms. Furthermore, patients need to be careful when taking medications such as anticoagulants to prevent the progression of the hematoma. Radiologic tests should be conducted regularly to monitor the size of the intraneural hematoma. If any symptoms surface, surgical treatment should be performed immediately.

A literature review was conducted to examine clinical data, the available diagnostic data, or treatment options of intraneural hematoma. de Ruiter et al 6 reported types of intraneural hematoma based on anatomical framework of nerve. Nerve layer anatomy was classified into paraneurium, epineurium, and intrafascicular layer (perineurium and endoneurium) and type of intraneural hematoma was also classified into subparaneurial, subepineurial, intrafascicular, and multicompartmental hematoma. For deciding adequate surgical treatment of intraneural hematoma, classifying clinical patient based on anatomy is essential.

Padua et al 12 described usefulness of ultrasonography on peripheral nerve system disease. In patient with peripheral nerve disease including compression of nerve due to intra- or extraneural hematoma, up to 82% of group could confirm or modify the diagnosis and therapeutic plan by ultrasonography. Arányi et al 13 also reported utility of ultrasonography on nerve injury by confirming intraneural neovascularization after penetrating nerve injuries. This indicates that high-resolution ultrasound can easily diagnose rare intraneural hematoma from other mass diseases. Rusu et al 14 also advocate Doppler ultrasonography as a first choice of diagnostic method on peripheral intraneural vascular anomalies disease. Even though MRI is widely regarded as a diagnostic gold standard for evaluating hemangiomas, 10 14 ultrasonography can also be a first choice of diagnosis for its usefulness of directly depicting intraneural location of abnormality and accurately differential diagnosing the anomalies of vessels. Therefore, concerning cost-effectiveness and accuracy of diagnosis, ultrasonography should be in an important role in diagnosis on intraneural hematoma or perineural vascular anomalies. However, for more accurate diagnosis, well-suited skills on ultrasonography are required on clinics by training. 12

There are some case reports without surgical excision of intraneural hematoma. Schwab et al 15 reported surgical exploration of intraneural hematoma without evacuation, due to diffuse spreading of hematoma with no relevant thickening of nerve. However, in most of intraneural hematoma, surgical evacuation was performed with good neurological outcomes. 1 5 7 de Ruiter et al 6 also described surgical treatment based on anatomical location of intraneural hematoma: paraneuriotomy, epineuriotomy with or without interfascicular neurolysis, or resection and reconstruction of affected nerve segment on severe case. However, owing to its rarity, comparison between surgical evacuation and conservative management cannot be made.

There are articles advocating surgical treatment through animal experiments. Rayan et al 16 and Scopel et al 17 reported that studies with rats have shown that intraneural hematoma-associated compression of nerve fibers causes axonal degeneration. Furthermore, surgical decompression is associated with a shorter functional recovery time. Therefore, on intraneural hematoma with compression and thickening of specific lesion of nerve, surgical evacuation might be better option than conservative management. Also, through future study, there should be consensus on severe cases that require segmental resection of nerve.
